# Bioavailability and Sustained Plasma Concentrations of CoQ10 in Healthy Volunteers by a Novel Oral Timed-Release Preparation

**DOI:** 10.3390/nu11030527

**Published:** 2019-02-28

**Authors:** Alessio Martucci, Delia Reurean-Pintilei, Anamaria Manole

**Affiliations:** 1 Ophthalmology Unit, Department of Experimental Medicine, University of Rome Tor Vergata, 00133 Rome, Italy; 2 Department of Diabetes, Nutrition and Metabolic Diseases, Consultmed Medical Center, 700547 Iasi, Romania; drdeliapintilei@gmail.com (D.R.-P.); anamaria_manole10@yahoo.com (A.M.)

**Keywords:** coenzyme Q10 (CoQ10), bioavailability, intestinal absorption, neuroprotection

## Abstract

Coenzyme Q10 (CoQ10) is a natural compound with potent antioxidant properties. Its provision through diet does not always allow adequate levels in the human body, and supplementation is often necessary. This bioavailability study intended to explore the plasma concentration levels of a novel CoQ10 oral preparation (COQUN^®^, Coenzyme Q10 Miniactives Retard 100 mg capsules) mimicking assumption on a regular basis. Twenty-four healthy adults tested a single dose of CoQ10 100 mg in one day to assess bioavailability. After a one week wash-out period, they were randomly assigned (1:1) to continuous administration for four weeks: Group A (*n* = 12) 100 mg once a day (OD); and Group B (*n* = 12) 100 mg twice a day (BID). During the single dose phase, C_max_ was observed at 4 h, and the mean values of AUC_t_ and T_max_ were 8754 μg/mL·h and 4.29 h, respectively. The multiple dose phase showed increasing plasma levels up to 7 days after the start of administration, and sustained high concentrations during the all administration period. No relevant adverse events were reported. These results show that Miniactives^®^ technology can release CoQ10 to allow high constant blood concentrations without a sharp decrease. This may be the first step of evidence for a potential new antioxidative treatment in human chronic diseases deserving high CoQ10 levels.

## 1. Introduction

Coenzyme Q (CoQ), or ubiquinone, is a lipophilic, vitamin-like compound with exceptional biochemical properties, synthetized by prokaryotic and eukaryotic cells. CoQ10 is the lipid form produced by the human body, where Q10 indicates the number of isoprenoid subunits in the lipid tail attached to the quinone ring of the coenzyme [1]. CoQ10 can also be obtained from diet, mainly from meat, poultry, and fish, and in much less quantity from fruits, vegetables, cereals, and dairy products [2]. 

CoQ10 is a physiological component of the human mitochondrial electron transport chain, but its half-reduced and fully reduced forms allow CoQ10 to function as an antioxidant [3]. By virtue of its proven ability to change in a reduced form, CoQ10 has been shown to induce protective effects against lipid peroxidation in a ubiquitous manner in the human body, with special regard in organs and systems’ tissues such as cardiovascular, nervous, and metabolic. Over the years, an ever-increasing number of diseases have been associated with mitochondrial dysfunction and oxidative stress.

Significant reduction of cardiovascular mortality, decrease of NT-proBNP blood levels, and improvement of cardiac function has been reported among elderly subjects after five years of combined supplementation of CoQ10 and selenium [4]. The long-term (two years) beneficial effects of CoQ10 supplementation on symptoms improvement and reduction of major adverse cardiovascular events (i.e., cardiovascular and all-cause mortality, and incidence of hospital stay) have also been assessed in patients with chronic heart failure (CHF) [5]. 

Most of clinical evidence sustains that the glycemic control among individuals with type-2 diabetes mellitus (T2DM) can be improved by CoQ10 supplementation. CoQ10 administration at different daily doses (ranging 60–200 mg/day) and for different periods (eight weeks–six months) can determine increased insulin synthesis and secretion by pancreatic β cells, significant decrease of glycated hemoglobin level, and kidney protection against diabetic nephropathy. However, despite other studies reported, marginal or not significant clinical benefits of CoQ10 in glycemic control, it is now clear that mitochondrial dysfunction is secondary to oxidative stress that, most of time, can be successfully treated by adequate supplementation of CoQ10 in T2DM patients [6].

Promising results have been reported in the treatment of neurodegenerative disorders such as Parkinson disease (PD) and Huntington’s disease (HD) with CoQ10. As these are chronic, progressive and non-regressive disorders, the goal of any treatment is to cause a slowing of the disease progression, since improvement and cure are not currently possible. That is why only the highest dose of CoQ10 slowed the functional decline of PD among the three dosages tested of 300, 600 or 1200 mg/day in subjects with the early stage of disease, and not yet requiring treatment for their disability [7]. Conversely, a chronic supplementation with 600 mg/day of CoQ10 did not produce any significant slowing in functional decline in patients with early HD [8]. 

An interesting field of CoQ10 neuroprotective research against reactive oxygen species focused on promoting mitochondrial functions and retinal ganglion cell (RGC) survival in ischemic retina under conditions of intraocular pressure elevation (glaucoma). Glaucoma is a progressive neurodegenerative disease of RGCs associated with axon degeneration in the optic nerve. During recent years, researchers became aware that traditional strategies of lowering intraocular pressure were often unsatisfactory to prevent progressive vision loss. Thus, the current trend of using neuroprotective strategies for the treatment of glaucoma is sustained by the growing evidence that glaucomatous neurodegeneration is analogous to other neurodegenerative disorders in the central nervous system [9,10]. Consistently, CoQ10 showed to significantly block activation of astroglial and microglial cells and apoptosis in ischemic retina in addition to protecting RGCs in animals [11], and to improving inner retinal function and visual cortical responses in humans [12]. 

Combination of appropriate formulations and dosages is a key factor to allow optimal absorption and achieve adequate blood concentration of CoQ10 to exert the expected clinical benefits. The importance of achieving an optimal CoQ10 bioavailability is justified by the possible risk of exposing treated subjects to a lack of efficacy in the case of underdosing. On the other hand, too high concentrations can induce toxic effects or increase the rate of adverse events. The clinical evidence suggests that CoQ10 bioavailability can greatly vary not only after different daily doses or dose strategies, but especially belongs to formulations used [13,14,15,16,17]. For instance, it was shown that an emulsified CoQ10 preparation can increase the intestinal absorption, being more permeable across cellular membranes and allowing a relatively low-dose administration [13]. Despite many other factors can influence plasma CoQ10 concentrations, such as serum lipoproteins levels, i.e., cholesterol, High Density Lipoprotein (HDL), and Low Density Lipoprotein (LDL)/Very Low Density Lipoprotein (VLDL) are carriers of CoQ10 in the circulation–diet, daily motion, time of day, human race, age, and gender, and some authors indicated that dissolution is probably the more important factor rather than release and absorption rate [14,15]. Lu and coworkers [14] administered the same single daily dose of CoQ10 (50 mg/day) to a small group of healthy Asian volunteers using two different formulations. The baseline plasma values, and after day 15 of treatment of CoQ10, were similar to the respective values observed in European subjects, but CoQ10 bioavailability was higher in subjects treated with the sustained release tablets compared to regular tablets. Another colloidal CoQ10 preparation achieved astonishingly higher plasma levels compared to the same doses (120 mg/day) of other more conventional formulations [15].

Good intestinal absorption and the achievement of high peak plasma concentrations should not be the only objectives of an oral formulation of CoQ10. It is important to ensure that plasma concentrations remain constant over time, avoiding excessive fluctuations in bioavailability, especially if once-daily dosing is clinically required. The authors of Reference [16] showed that the plasma concentrations of the five formulations used, following a high peak reached after 2–4 h, returned to the same initial levels after 12 h from the administration. Moreover, the dosing strategy is another major important cause to reach adequately high plasma CoQ10 concentrations. A divided dose administration (e.g., BID) improves absorption by almost double, as compared with the same amount of active substance taken in one single dose [16]. The dose fractionation strategy should be carefully considered when high doses have to be administered or when high bioavailability should be achieved with a relatively small daily dose. 

The aim of this bioavailability study was to determine the single (100 mg) and multidose dose (100 mg/day vs. 2 × 100 mg/day) pharmacokinetics (i.e., dosage effect and dosage strategy) of a novel CoQ10 preparation based on neutral micro-particles dissolution technology (i.e., formulation effects), a prolonged-release capsule administered orally to healthy volunteers. Bioavailability intended to explore the plasma concentration levels which might assure antioxidant effect if the novel CoQ10 preparation were taken on a regular basis.

## 2. Materials and Methods

### 2.1. Study Design

This was a single-center (Consultmed Iasi, IASI, Romania), open-label, single and multi-dose bioavailability study of an innovative CoQ10 oral formulation in 24 healthy adults. All subjects tested a single dose of 100 mg of CoQ10 in 1 day to assess bioavailability. Then, the subjects followed a 1 week wash-out period after which they were randomly assigned (1:1) to a 4 week period of continuous administration of CoQ10: Group A of 12 subjects with intake of 100 mg OD (after dinner); Group B of 12 subjects with intake of 100 mg BID (after lunch and dinner). The primary objective was to evaluate the best dosage between 100 mg OD or 100 mg BID of the novel CoQ10 oral formulation in order to reach a level of plasma concentration which might assure its antioxidant effect if taken on a regular basis. The secondary objectives were to evaluate the safety and tolerability of both the single 100 mg oral dose and the multiple doses of 100 mg OD and BID during the 1 month daily dose phase.

In order to participate in the study, each subject had to meet all major inclusion and exclusion criteria at screening and at check-in visits. Inclusion criteria: informed consent form (ICF) signed, both gender aged between 25–75 years, body mass index (BMI) between 20–29 kg/m^2^, fasting the night before enrolment for at least 10 h, healthy status, abstention from consumption of any food supplements except vitamin D and calcium at least 2 weeks before and during the study, consumption of dairy and cereal products, and willing to follow all study procedures. Exclusion criteria: intake of any prescribed medication within 2 weeks of the beginning of the study, hypotension, any clinically significant history of serious digestive tract, liver, kidney, cardiovascular or hematological disease, diabetes, gastrointestinal disorders, or other serious acute or chronic diseases, known lactose/gluten intolerances/food allergies, inadequate veins, or known contraindication to placement of a dedicated peripheral line for venous blood withdrawal, known drug and/or alcohol abuse, use of any form of nicotine or tobacco, mental incapacity precluding adequate understanding or cooperation, participation in another investigational study or blood donation within 3 months prior to or during this study. 

During the study the following procedures were performed: Physical examination, vital signs recording (blood pressure, heart rate, temperature, and respiratory rate), body measurements (height and weight), 12 lead electrocardiogram (ECG), safety laboratory analysis (Haematology: Red blood cells, white blood cells, platelet, haemoglobin, and haematocrit; Biochemistry: hepatic transaminases, alcalin phosphatase, total cholesterol, LDL, and HDL cholesterol), concomitant medication recording, and adverse events monitoring. With special regard to the latter, mild insomnia, elevated levels of liver enzymes, rash, nausea, upper abdominal pain, dizziness, sensitivity to light, irritability, headache, heartburn, and fatigue were closely monitored.

### 2.2. Pharmacokinetic Timing and Assessments

When they arrived at the study center, subjects were hospitalized for at least 12 h for the single dose phase, and they remained at study site for approximately 24 h. At the end of this visit (visit 1), if no serious adverse event (SAE) occurred, subjects were dismissed and requested to return after 1 week (wash-out period) for the second study visit (visit 2), in order to initiate the multidose phase. Besides, subjects received a diary and were requested to report any possible adverse event experienced between V1 and V2, as well as any treatment taken for treating adverse events (AEs), if applicable. In the wash-out period subjects had to respect the same lifestyle regimen and no medication had to be taken, if not necessary. 

**Figure 1:**
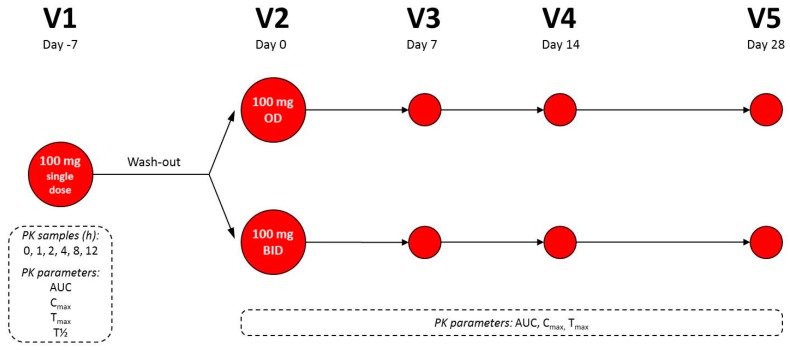
Flow-chart of blood sampling times (⬤) for the bioavailability assessment after a single dose of 100 mg and after multiple doses of 100 mg once a day (OD) or twice a day (BID) of CoQ10. During visits, V2, V3, V4, and V5 (multiple dose phase) only had one sample (at one timepoint) was collected. AUC: area under the curve; PK: pharmacokinetic; Cmax: maximum concentration; Tmax: the time at which the Cmax is observed; T1/2: half-life.

During the single-dose phase plasma CoQ10 levels were measured before dosing (0 h) and over the next 12 h after intake: at 1, 2, 4, 8, and 12 h. Pharmacokinetic properties were measured accordingly: Area under the curve until the last observation (AUC_t_) (μg/mL·h), maximum plasma concentration (C_max_) (μg/L), time at which the C_max_ was observed (T_max_) (hours), and elimination half-life (T_½_) (hours). During the multidose-dose phase, plasma CoQ10 concentrations were measured once at the following timepoints: V2 (day 0), V3 (day 7), V4 (day 14), and V5 (day 28) (Figure 1). The pharmacokinetic properties measured were AUC_t_, C_max_, T_max_. Samples were analyzed for plasma CoQ10 using an immunosorbent assay (ELISA; enzyme-linked immunosorbent assay) validated at the analytical laboratory (Consultmed Iasi Laboratory, IASI, Iasi county, Romania).

For pharmacokinetic analyses 4 mL of blood were collected in blood collection tubes. Each blood sample was allowed to clot for 20–25 min at room temperature. Then, they were centrifuged for 15 min at 1300 g at 4 °C. Afterwards the plasma was separated into the secondary sample tubes as follows: 0.5 mL plasma into two cryotubes, one to be sent to the pharmacokinetic laboratory and one as back-up. The plasma cryotubes were appropriately labeled (study code, treatment period, subject number, sampling time) and stored at −20 °C to −80 °C at the study center until shipment to the specified laboratory. The backup cryotubes were kept at the study center at least until the confirmation from the pharmacokinetic laboratory that the samples arrived in good conditions.

### 2.3. Formulation Administered

The CoQ10 formulation administered (COQUN^®^, Coenzyme Q10 Miniactives Retard 100 mg capsules, Visufarma S.p.A.) is a novel oral preparation based on an innovative modified release technology of active principles at certain time intervals. The basis in Miniactives^®^ form is neutral microparticles of round shape, with dimensions between 400 and 500 microns. Each single particle is covered with one or more concentric layers of the active ingredients, and subsequently coated with a polymeric membrane suitable for obtaining a pre-established timed release. This technology leaves the time of the active ingredients absorption unchanged. This formulation gradually releases the active ingredients by diffusion, in a pre-determined time, thanks to a polymeric permeable and insoluble membrane coating each single particle, thus assuring a constant release.

### 2.4. Ethical Conduct of the Study

All subjects gave their informed consent for inclusion before they participated in the study. The study was conducted in accordance with the Declaration of Helsinki revised in 2013, and the protocol was approved by the local Ethics Committee Comisia Locala de Etica Consultmed, Sos Paracurari n. 70, bl 550, parter, Iasi, Romania (Project identification code: VF-BAQ10/2018) on May 31st, 2018, and also approved by Romanian Ministry of Health (Ministerul Sanatatii, Str Cristian Popisteanu, *n*. 1–3, Bucaresti, Romania) on June 19th, 2018. 

The study was registered at ClinicalTrials.gov (Internet). Bethesda (MD): National Library of Medicine (US) (Identifier NCT03819491).

### 2.5. Statistical Analysis

Due to the explorative aim of the study no formal power calculation has been attempted, and no hypotheses were pre-specified. Twenty-four subjects (12 for each arm) have been considered sufficient to obtain reliable results for the exploratory purposes of the study. Descriptive statistics and confidence intervals (CI) at 95% level are provided. In particular, continuous variables are presented as arithmetic mean values ± standard deviation (SD), median values with interquartile range, minimum, maximum, and coefficient of variation (CV); for categorical variables, the absolute and percentage frequencies are provided. When normality assumption hold, results of Student *t*-tests are presented in order to compare pharmacokinetics parameters in subjects assigned to the two treatment groups. The statistical software package used was SAS version 9.4.

The following analysis sets were considered in the study: Safety Population (SP), all randomized subjects who signed the informed consent and took at least one dose of study product; Intention-To-Treat Population (ITT), all randomized subjects; and Protocol Population (PP), all subjects who met all inclusion/exclusion criteria and who did not have any major protocol deviation.

## 3. Results

Twenty-seven subjects were screened in the study but 3 were screening failures. A total of 24 subjects entered the study, half (*n* = 12) allocated to CoQ10 100 mg OD oral intake and half (*n* = 12) to CoQ10 100 mg BID. All randomized subjects completed the study. The SP, ITT- and PP-population were composed of 24 subjects. The two treatment groups were comparable for baseline characteristics (Table 1). A complete listing of all demographic variables of each participating subject is reported in Table S1.

### 3.1. Single Dose Phase

**Figure 2:**
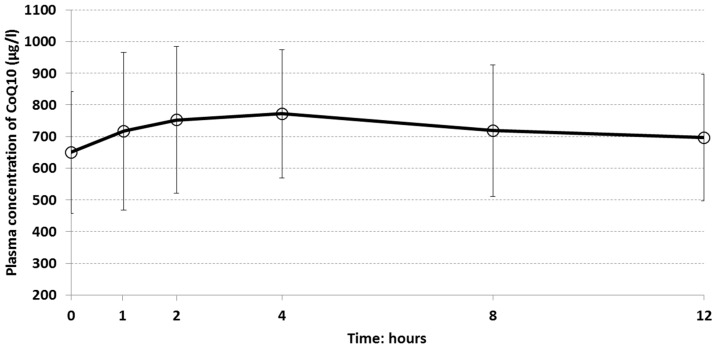
Single dose phase: mean plasma concentrations of CoQ10 by time after 100 mg single dose oral administration— Intention-To-Treat Population (ITT) population.

The plasma concentration curve of CoQ10 over time is shown in Figure 2. The distribution of plasma concentrations was wide as well as standard deviations, indicating quite different levels of plasma concentration among subjects. The mean baseline plasma CoQ10 concentration (0 h) was 649.8 (191.8) μg/L. However, a slightly increasing absorption phase of CoQ10 mean plasma values was observed until 4 h (772.1 μg/L), followed by a slow terminal decline until 12 h (696.3 μg/L) (Table S2A. Plasma concentration of CoQ10 by time (0, 1, 2, 4, 8, 12 h) during the single dose phase (V1)—ITT population; Table S2B. Individual plasma concentrations of CoQ10 (μg/L) in the single dose oral administration phase—ITT population).

The descriptive statistics for pharmacokinetic parameters in the single dose phase are reported in Table 2. The mean AUC_t_ was 8754 μg/mL·h. The maximum registered value was almost the double the mean (15,394.54 μg/mL·h) while the minimum was 5277.62 μg/mL·h. Among other parameters, T_max_ values showed very high fluctuation with a minimum of 0 h (i.e., the maximum concentration was reached before CoQ10 oral intake) and a maximum of 12 h. The standard deviation was 3.58 h and the coefficient of variation was 83%, indicating that subjects registered very different time of maximum concentration (Table S3. Individual pharmacokinetic parameters of CoQ10 in the single dose oral administration phase—ITT population). 

Males showed higher mean plasma concentrations compared to females at each time point (i.e., males: 759.79, 894.74, 844.33, 915.58, 840.01, and 772.41 μg/mL; females: 594.79, 628.38, 706.88, 700.34, 657.84, and 658.28 μg/mL, at 0, 1, 2, 4, 8, and 12 h, respectively). In the samples, significantly different plasma concentrations of CoQ10 between males and females were registered (*p* < 0.001); on average, females had a plasma concentration of 182.16 µg/L lower than men.

### 3.2. Multiple Dose Phase

**Figure 3:**
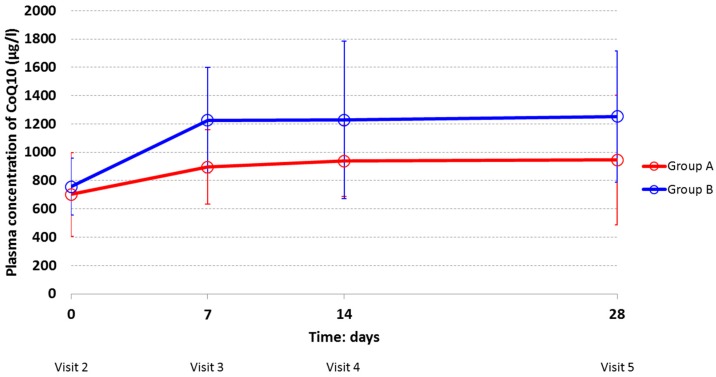
Multiple dose phase: plasma concentrations of CoQ10 by visit and by treatment group (Group A: 100 mg OD; Group B: 100 mg BID)—ITT population.

The mean plasma concentrations of CoQ10 increased over time in both treatment groups (Figure 3). At each study visit subjects who were assigned to 100 mg BID (Group B) had higher mean values than subjects in Group A, indicating that the assumption CoQ10 twice a day gave a higher concentration in the body.

The two dosages of the same CoQ10 oral formulation followed the same pattern over time. In both groups, the mean values of plasma concentrations increased considerably from Visit 2 (Day 0) to Visit 3 (Day 7). At Visit 2 (Day 0), the mean plasma CoQ10 concentrations in Group A and B (701.95 ± 295.17 μg/L and 756.96 ± 201.17 μg/L, respectively) represent the mean plasma values after the end of the washout period. After day 7 (Visit 3), constant trends of high plasma levels were observed, which remained high during the rest of the multidose phase (21 days), with higher values for the Group taking 100 mg BID.

Additionally, in the multidose phase males showed higher plasma concentration of CoQ10 compared to females at each study visit. As in the single dose phase, the pattern over time was the same for men and women. The distribution of plasma concentration was very wide: Standard deviations and coefficients of variation—showing the extent of variability in relation to the mean of the population—were quite large indicating that levels of plasma concentration were different among individuals. The high inter-individual variability observed in this study could be probably due to a series of physiological conditions such as age, gender, and multiple administration time, which makes a subject very different from another. More likely, by reviewing the pharmacokinetics profile of each subject randomized in this study, exceptionally high pharmacokinetic values were observed. They belong to a 36-year-old man randomized to Group B, who registered values of plasma concentration far above from the mean of the total set of subjects. By considering this individual as an outlier and by excluding him from the analysis (*n* = 23), mean plasma concentration values (and SD) were smaller for Group B, as follows (µg/L): 743.83 (205.53), 1153.86 (290.86), 1118.12 (421.44), and 1155.94 (338.48) at visit 2, visit 3, visit 4, and visit 5, respectively (Table S4. Individual plasma concentrations of CoQ10 (μg/mL) in the multiple dose oral administration phase—ITT population). 

The descriptive statistics for pharmacokinetic parameters by treatment group are reported in Table 2. The mean CoQ10 bioavailability (AUC_t_) in Group B (3459.05 µg/mL·h) was statistically higher than in Group A (2657.45 µg/mL·h) (*p* = 0.0345). Despite values of the other pharmacokinetics parameters (C_max_, T_max_) remained higher for subjects treated by CoQ10 100 mg BID compared to ones with the OD dosing scheme, no statistically significant differences between groups were detected. As previously described, by excluding the outlier subject from the analysis, the difference between the AUC_t_ values of the two groups was no more statistically significant (*p* = 0.0548) (Table S5. Individual pharmacokinetic parameters of CoQ10 in the multiple dose oral administration phase—ITT population). In the scenario without this subject, there is evidence of a difference in the CoQ10 bioavailability (AUC_t_) between the two dosages of the novel oral formulation of CoQ10, but the low power of the study due to the small sample size did not allow highlighting, albeit slightly, a significant difference when excluding him.

### 3.3. Adverse Events

The oral formulation of CoQ10 was well tolerated in all 24 healthy subjects; only 3 non-serious, moderate intensity AEs were reported during all the study period. The 3 AEs occurred in 2 subjects, both enrolled in the Group B (100 mg BID): (1) Intermittent dizziness of 5 days duration, possibly related to the oral preparation, and spontaneously resolved; (2) 1 day respiratory virosis, adequately treated, but unrelated to the study product; and (3) pultaceous angina of 5 days duration, unrelated to the CoQ10 oral preparation.

During the multiple dose phase (at visit 2 and visit 5), 21 subjects (9 in Group A and 12 in Group B) showed some biochemical values out of normal ranges, but none was considered clinically significant. Only 5 subjects—2 in Group A and 3 in Group B—showed clinically not significant out-of-range liver biochemical values. The ranges of values considered outside the normal ranges are shown in Table 3. 

Subjects randomized to treatment with 100 mg BID of CoQ10 did not show higher out-of-range liver values compared to subjects treated with the halved dose (100 mg OD). Both doses were safely tolerated. However, renal function tests were not monitored during the study because they were not included in the protocol requirements, and no blood samples were taken for the evaluation of basic plasma biochemistry. 

## 4. Discussion

These results demonstrate that in human plasma high levels of CoQ10 can be achieved by administrating relatively low oral doses by the use of a novel timed-release oral formulation determining optimal intestinal absorption and sustained plasma concentrations over time. The combination of bioavailability and safety results obtained with two oral dosages of CoQ10 (100 mg OD and BID) contribute to the construction of a rationale for a clinical use of this novel formulation of CoQ10. The information can help clinicians to protect patients from the negative effects of lipid peroxidation, on the one hand preventing possible therapeutic failures due to CoQ10 underdosing, and on the other, the possible development of toxicity following administration of too high doses.

In the experience of Joshi and coll. the pharmacokinetic properties of two new oral CoQ10 formulations (i.e., fast-melting tablet and effervescent tablet) were not statistically different compared with those of commercial formulations (i.e., soft gelatin capsule and powder-filled hard shell) when administered at 60 mg in single dose fashion [16]. The mean C_max_ values of the four formulations (around 80 μg/mL) measured over 12 h were essentially similar to the one of CoQ10 Miniactives Retard capsule we studied (83 μg/mL), but the mean T_max_ values were almost halved (1.3 and 2.0 h for fast-melting and effervescent tablets, respectively) compared to the one of Miniactives capsule (4.29 h). It is hard to believe that the more rapid delivery of the two fast melting formulations can play a significant role in the clinical cure of diseases in which the main feature of long-term treatment should be to ensure consistently high levels of CoQ10 over time. Furthermore, the bioavailabilities of the four formulations (ranging 4.9–5.5 μg/mL·h) were far below the bioavailability of the one we tested (8.754 μg/mL·h). This can be probably explained mainly by the dissolution properties of the Miniactives^®^ technology that allows more sustained plasma concentrations over time (until 12 h after dosing), as well as by the higher dose administered (100 mg) in our study. 

When supplemented at 100 mg/day by oral formulation consisting of soya oil in soft gelatin capsule (Myoqinon^®^ 100 mg CoQ10), CoQ10 achieved median plasma concentration of 2.5 mg/L (2500 μg/L) after a 2 month administration period [18], far above the median plasma level achieved at day 28 with Miniactives capsule (773.35 μg/L) administered at 100 mg/day in Group A. It is difficult to draw conclusions when comparing our results with those of Zita and coll. [18] due to the profound difference between the durations of oral administration of CoQ10 in the two studies (2 months vs. 1 month). In addition to this, no other pharmacokinetic parameters were reported by the authors of Reference [18]. Astonishingly, in another study [17], the same 100 mg/day CoQ10 soya oil in soft gelatin capsule (Myoqinon^®^ 100 mg CoQ10) did not generate comparable pharmacokinetic results to those reported by Zita. After 20 days CoQ10 plasma concentration was approximately 0.9 mg/L (900 μg/L), a result much below the 2.5 mg/L reported in the study [18]. The mean plasma concentrations of CoQ10 after administration of oil/soft gel formulation used by Singh [17] are closer to those observed in our study after 28 days of CoQ10 100 mg/day administration (944.8 μg/L). Moreover, the author also highlighted the importance of the dosing strategy in addition to the daily dose. Divided dosages (2 × 100 mg) of oil/soft gel CoQ10 formulation caused a larger increase in plasma levels of CoQ10 (approximately >1.9 mg/L) than a single dose of 200 mg (approximately >1.3 mg/L) [17]. Our results show that, after 28 days of supplementation of Miniactives^®^, formulation 2 × 100 mg the mean plasma concentration was a little higher than 1200 μg/L. 

With the aim to overcome the poor intestinal absorption, the bioavailability of a CoQ10 colloidal oral preparation was determined versus one oil-based formulation and two solubilizates in a single dose (120 mg) study. The mean C_max_ colloidal formulation was the highest among the four formulations studied (6890 μg/L), as well as its AUC_(0-10)_ (30,620 μg/mL·h). Nevertheless, the oil-based formulation and the two solubilizates showed rather high bioavailability (i.e., 4900, 6100, 10,700 μg/mL·h, respectively) [15]. When comparing the pharmacokinetic profiles of the four formulations with Miniactives^®^ capsule, all achieved the peak of plasma concentration 4 h after the administration and maintained sustained levels afterwards. Once again, it is difficult to compare the results of different studies, since several factors may have contributed to the achievement of a particular bioavailability profile (e.g., study design, selection criteria, diet, analytical procedures, etc.). In the case of colloidal preparation, it is undoubted that it has favored the intestinal absorption of the conveyed CoQ10. 

Very recently, a pharmacokinetic study highlighted the importance of inter-subjects variability in the plasma level of CoQ10 caused by significant variation of intestinal absorption of CoQ10 between subjects and irrespective of the oral formulation or molecular form administered [19]. The three commercial preparations tested (i.e., ubiquinol 150 mg capsule, ubiquinone 150 mg capsule and liposome ubiquinone 40 mg/2 sprays) showed plasma levels of CoQ10 ranging 5000–6000 μg/L at the 2 h interval, with ubiquinol preparation having the highest response, but a high inter-individual variation was observed for each preparation at every time interval. In our experience, this phenomenon has also been observed in both the single and multiple dose phase. After the single dose phase, the mean AUC_t_ (8754.34 ± 2382.03 μg/mL·h) showed a very wide range (5277.62–15,394.54 μg/mL·h) and also mean T_max_ had a high fluctuation, indicating different times of maximum concentration between subjects (coefficient of variation: 83%). During the multidose phase a very wide distribution of plasma concentration was observed, indicating quite different levels among individuals. Particularly, the exceptionally high pharmacokinetic values of a single subject (36-year-old man) randomized in the group of 2 × 100 mg CoQ10 dose contributed to increase in the overall variability of pharmacokinetic results of the entire subject population. Despite the bioavailability values of CoQ10 of the two groups (Group A and Group B), they were not statistically different after exclusion of the outlier subject (*p* = 0.0548), however, there is evidence of a difference at limits of the significance threshold (*p* < 0.05) between the two groups. Probably, a clear difference did not emerge due to the small sample size studied.

During both single and multiple dose phases, males showed higher plasma CoQ10 concentration than females at each time point. CoQ10 baseline is naturally higher in men than in women [14,20], ranging 0.40–1.72 μmol/L (345.34–1484.94 μg/L) for males and 0.43–1.47 μmol/L (371.24–1269.11 μg/L) for females in European (Finnish) population [20]. Our results on baseline CoQ10 plasma levels (0 h) in both sexes are included in these normal ranges (males 759.79 ± 198.09; females 594.79 ± 168.63). During the multidose phase, the same pattern of plasma CoQ10 concentration was observed between genders (e.g., males 909.79 ± 241.54 μg/L, females 639.29 ± 203.81 μg/L at Visit 2; males 1173.32 ± 524.40 μg/L, and females 1061.03 ± 464.98 μg/L at Visit 5). In this study, the proportion between genders was unbalanced (i.e., 8 males/16 females). However, this disproportion did not appear to have influenced the plasma concentrations of CoQ10 according to the expected levels in males and females. In addition, another pharmacokinetic study reported the same numerical disproportion between the two genders, without any reported influence on the observed results [15]. Regarding the difference between genders in CoQ10 plasma concentrations, our results are in line with previous studies. The present experience and others in the literature support the conclusions that men can have better absorption and/or lower clearance than women [13].

The results presented in our bioavailability study suggest that Miniactives^®^ timed release formulation of CoQ10 gradually released the active ingredient by diffusion, in a pre-determined time, thanks to a polymeric permeable and insoluble membrane coating each single particle, thus assuring a constant release. After having achieved the peak at 4 h, CoQ10 plasma concentrations did not undergo a sharp decrease and remained constantly high. The development of this technology was supported with the aim of creating an oral formulation able to ensure consistently high CoQ10 blood concentrations, useful for supporting a treatment strategy in the neuroprotection of RGC. In glaucoma, retinal neuroprotection can be significantly improved through maintenance of mitochondrial functions and survival of RGC by CoQ10, one of the most powerful antioxidant compounds. The potential clinical significance of this finding should be further evaluated.

In conclusion, based on the obtained results and on data available in literature regarding the expected average plasma levels of CoQ10, this exploratory study highlighted that both 100 mg OD or BID are safe and assure a plasma concentration of CoQ10 that remains high for the duration of the intake and that 100 mg COQUN^®^ Miniactives^®^ BID would be preferred than OD in reaching a higher plasma concentration of CoQ10. These positive results suggest that further studies are needed in order to investigate the antioxidative effects of COQUN^®^ OS oral formulation in patients with specific diseases like glaucoma where the antioxidative effect of the CoQ10 is expected to be seen at the target organ.

## Figures and Tables

**Table 1:** Demographic features of subjects randomized in each CoQ10 treatment group.

	Group A	Group B	Total
	(CoQ10 100 mg OD)	(CoQ10 100 mg BID)	
Gender			
Female *n* (%)	8 (66.67)	8 (66.67)	16
Male *n* (%)	4 (33.33)	4 (33.33)	8
Ethnic Group			
Caucasian *n* (%)	12 (100.00)	12 (100.00)	24
Age (years)			
Mean (SD)	35 (7.08)	43 (6.61)	39 (7.69)
Median	35	43	39
Range	26–46	30–50	26–50
Height (cm)			
Mean (SD)	168.08 (5.85)	165.92 (8.51)	167.00 (7.23)
Median	169.50	162.50	167.00
Range	156.00–178.00	154.00–185.00	154.00–185.00
Weight (kg)			
Mean (SD)	70.88 (9.59)	70.24 (13.87)	70.56 (11.67)
Median	71.50	76.20	72.50
Range	58.00–85.00	48.00–87.00	48.00–87.00
BMI (kg/m^2^)			
Mean (SD)	25.05 (2.84)	25.16 (3.37)	25.11 (3.05)
Median	25.02	25.80	25.47
Range	20.79–28.81	20.23–28.86	20.23–28.86

SD: standard deviation.

**Table 2:** Descriptive statistics for pharmacokinetic parameters of CoQ10 in the single and multiple phase—ITT population.

Pharmacokinetic Parameters		Single Dose Phase (*n* = 24)	Multiple Dose Phase	
			Group A 100 mg OD (*n* = 12)	Group B 100 mg BID (*n* = 12)
AUC_t_ (μg/mL·h)	Mean (SD)	8754.34 (2382.03)	2657.45 (681.74)	3459.05 (1026.66)
	CI 95%	7748.41–9760.1	2224.29–3090.6	2806.75–4111.36
	Median	8319.91	2490.96	3520.28
	Range	5277.62–15,394.54	1671.77–3778.37	2173.31–6068.15
	Coefficient of variation (%)	27	26	30
	Interquartile range	2920.10	1025.78	1073.09
C_max_ (μg/L)	Mean (SD)	828.92 (233.09)	1163.99 (354.95)	1501.89 (474.64)
	CI 95%	730.49–927.35	938.46–1389.51	1200.32–1803.47
	Median	830.37	1131.38	1495.78
	Range	482.54–1438.45	679.81–1826.85	906.43–2441.84
	Coefficient of variation (%)	28	30	32
	Interquartile range	283.69	542.84	759.72
T_max_ (h */day **)	Mean (SD)	4.29 (3.58)	17.5 (10.12)	16.92 (8.68)
	CI 95%	2.78–5.80	11.07–23.93	11.4–22.43
	Median	4.00	14.00	14.00
	Range	0–12	0–28	7–28
	Coefficient of variation (%)	83	58	51
	Interquartile range	2.50	17.50	17.50
T_½_ (h)	Mean (SD)	8.88 (2.40)		
	CI 95%	7.87–9.90		
	Median	8.14		
	Range	6.77–16.26		
	Coefficient of variation (%)	27		
	Interquartile range	0.79		

* h: Tmax during single dose phase, ** day: Tmax during multiple dose phase.

**Table 3:** Range of values of liver biochemical parameters outside the normal ranges observed during the multiple dose phase in both treatment groups—ITT population.

Biochemistry (Normal Ranges)	Group A		Group B	
	(*n*. of Subjects) *		(*n*. of Subjects) *	
AST (<32 U/L)	39–42	(2)	34–40	(2)
ALT (<33 U/L)	43–48	(2)	42–96	(3)
ALP (35–104 U/L)	---		27–237	(2)
TC (<200 mg/dL)	203–235	(3)	200–251	(5)
HDL (≥60 mg/dL)	39	(1)	36–40	(2)
LDL (<100 mg/dL)	100–186	(8)	101–200	(11)
TGs (<100 mg/dL)	---		257	(1)

AST = Aspartate Aminotransferase; ALT = Alanine Aminotransferase; ALP = Alkaline Phosphatase; TC = Total Cholesterol; HDL = High-Density Lipoprotein; LDL = Low-Density Lipoprotein; and TGs = Triglycerides. * Some subjects experienced more than one biochemical value outside the normal range of the reference laboratory.

## Supplementary Materials

The following are available online at https://www.mdpi.com/2072-6643/11/3/527/s1, Table S1: Individual demographic variables—ITT population; Table S2A. Plasma concentration of CoQ10 by time (0, 1, 2, 4, 8, 12 h) during the single dose phase (V1)—ITT population, Table S2B. Individual plasma concentrations of CoQ10 (μg/L) in the single dose oral administration phase—ITT population; Table S3. Individual pharmacokinetic parameters of CoQ10 in the single dose oral administration phase—ITT population; Table S4. Individual plasma concentrations of CoQ10 (μg/L) in the multiple dose oral administration phase—ITT population; Table S5. Individual pharmacokinetic parameters of CoQ10 in the multiple dose oral administration phase—ITT population.
