# Supplementary material for: Bioavailability and Sustained Plasma Concentrations of CoQ10 in Healthy Volunteers by a Novel Oral Timed-Release Preparation
Source: Nutrients. 2019 Feb 28;11(3):527. doi: 10.3390/nu11030527 (PMC6471387; doi:10.3390/nu11030527)
Supplement: Supplementary file 1 [file nutrients-11-00527-s001.pdf]

Table 1. Individual demographic variables—ITT population.

Screening Number	Treatment Group	Date of assessment	Age (years)	Gender	Ethnic Group	Height (cm)	Weight (kg)	BMI (kg/m²)
01-02	A	03/07/2018	26	Male	Caucasian	178	73.0	23.04
01-05	B	05/07/2018	43	Female	Caucasian	162	57.0	21.71
01-06	A	05/07/2018	41	Female	Caucasian	164	77.5	28.81
01-07	B	05/07/2018	38	Female	Caucasian	154	48.0	20.23
01-08	A	05/07/2018	40	Female	Caucasian	156	62.0	25.47
01-09	B	05/07/2018	36	Female	Caucasian	163	59.0	22.20
01-10	A	05/07/2018	41	Female	Caucasian	166	72.0	26.16
01-11	B	05/07/2018	49	Female	Caucasian	162	75.4	28.73
01-12	A	05/07/2018	34	Female	Caucasian	173	85.0	28.40
01-13	B	05/07/2018	49	Female	Caucasian	171	84.0	28.72
01-14	A	12/07/2018	46	Female	Caucasian	161	58.0	22.37
01-15	B	12/07/2018	30	Female	Caucasian	160	54.0	21.09
01-16	A	12/07/2018	41	Female	Caucasian	167	58.0	20.79
01-17	B	12/07/2018	47	Female	Caucasian	160	58.0	22.65
01-18	A	12/07/2018	28	Female	Caucasian	170	71.0	24.56
01-19	B	12/07/2018	36	Male	Caucasian	175	80.0	26.12
01-20	A	18/07/2018	36	Female	Caucasian	173	82.0	27.39
01-21	B	18/07/2018	42	Male	Caucasian	185	87.0	25.47
01-22	A	18/07/2018	26	Male	Caucasian	170	65.0	22.49
01-23	B	18/07/2018	50	Female	Caucasian	160	77.0	28.12
01-24	A	18/07/2018	28	Male	Caucasian	170	83.0	28.71
01-25	B	25/07/2018	37	Male	Caucasian	172	83.0	28.05
01-26	A	25/07/2018	29	Male	Caucasian	169	64.0	22.40
01-27	B	25/07/2018	48	Male	Caucasian	167	80.5	28.86

Table S2A. Plasma concentration of CoQ10 by time (0, 1, 2, 4, 8, 12 hours) during the single dose phase (V1)—ITT population

	Plasma Concentration of CoQ10 at Visit 1 (μg/l)					
	Hour 0	Hour 1	Hour 2	Hour 4	Hour 8	Hour 12
Subjects (N.)	24	24	24	24	24	24
Mean (SD)	649.8 (191.8)	717.2 (248.8)	752.7 (231.5)	772.1 (202.2)	718.6 (207.6)	696.3 (200.2)
CI 95%	568.8-730.8	612.1-822.2	655-850.4	686.7-857.5	630.9-806.2	611.8-780.8
Median	642.3	689.7	744.8	728.6	699.5	685.2
Range	255.4-1168.1	344.5-1438.5	376.4-1242.2	450.8-1353.8	403.1-1256.2	406.6-1211.3
Coefficent of Variation (%)	30	35	31	26	29	29
Interquartile Range	171.7	311.7	275	281.8	250.3	272.4

Table S2B. Individual plasma concentrations of CoQ10 (μg/L) in the single dose oral administration phase—ITT population

Pt. N.	Gender	Age	Group	Visit 1					
				Hour 0	Hour 1	Hour 2	Hour 4	Hour 8	Hour 12
01-02	M	26	A	649.98	780.50	644.23	718.59	703.19	579.86
01-05	F	43	B	743.36	860.79	922.97	847.07	860.83	696.65

01-06	F	41	A	886.07	901.01	1239.09	845.43	951.66	794.53
01-07	F	38	B	569.30	864.10	781.94	687.69	706.68	614.14
01-08	F	40	A	435.90	627.35	543.15	632.63	573.15	616.39
01-09	F	36	B	647.99	604.90	608.54	584.62	645.50	537.37
01-10	F	41	A	598.12	357.63	619.04	738.64	617.32	702.19
01-11	F	49	B	572.86	344.48	796.65	708.42	695.77	673.74
01-12	F	34	A	733.28	498.27	828.76	889.22	789.38	818.75
01-13	F	49	B	844.29	820.79	806.90	933.34	731.64	895.91
01-14	F	46	A	255.43	442.02	376.40	544.29	403.12	522.54
01-15	F	30	B	572.52	886.61	897.65	883.42	846.34	925.04
01-16	F	41	A	438.43	453.71	402.87	450.83	420.70	482.54
01-17	F	47	B	348.71	534.33	526.87	566.76	450.05	483.26
01-18	F	28	A	636.64	635.31	719.43	602.39	646.75	722.10
01-19	M	36	B	633.88	906.23	1001.28	990.42	1106.55	1075.36
01-20	F	36	A	563.96	567.15	567.94	607.16	524.12	445.32
01-21	M	42	B	658.21	723.88	654.98	883.94	684.99	531.09
01-22	M	26	A	554.13	568.57	543.45	560.73	472.18	406.60
01-23	F	50	B	669.83	655.60	671.93	683.48	662.48	601.93
01-24	M	28	A	885.32	814.83	789.31	864.20	899.46	774.57
01-25	M	37	B	840.71	1117.90	1108.93	1050.15	844.67	872.81
01-26	M	29	A	688.03	807.55	770.27	902.76	752.83	727.72
01-27	M	48	B	1168.07	1438.45	1242.17	1353.84	1256.19	1211.26

Table S3. Individual pharmacokinetic parameters of CoQ10 in the single dose oral administration phase—ITT population

Pt. N.	AUC_t (μg/ml·h)	C_{max} (μg/l)	T_{max} (hours)	Dose (mg)	Clearance	Distribution Volume	$T_{½}$ (hours)
01-02	8200.09	780.50	1	100	0.012	0.128	7.282
01-05	9994.76	922.97	2	100	0.010	0.116	8.048
01-06	11134.67	1239.09	2	100	0.009	0.111	8.566
01-07	8439.73	864.10	1	100	0.012	0.116	6.770
01-08	7083.30	632.63	4	100	0.014	0.159	7.826
01-09	7252.31	647.99	0	100	0.014	0.165	8.310
01-10	7674.83	738.64	4	100	0.013	0.280	14.875
01-11	8081.71	796.65	2	100	0.012	0.290	16.262
01-12	9570.73	889.22	4	100	0.010	0.201	13.314
01-13	9971.69	933.34	4	100	0.010	0.122	8.421
01-14	5424.77	544.29	4	100	0.018	0.226	8.507
01-15	10405.05	925.04	12	100	0.010	0.113	8.135
01-16	5277.60	482.54	12	100	0.019	0.220	8.063
01-17	5965.99	566.76	4	100	0.017	0.187	7.739
01-18	7871.15	722.10	12	100	0.013	0.157	8.588
01-19	12273.27	1106.55	8	100	0.008	0.110	9.387
01-20	6509.64	607.16	4	100	0.015	0.176	7.956
01-21	8489.42	883.94	4	100	0.012	0.138	8.129
01-22	6044.92	568.57	1	100	0.017	0.176	7.369
01-23	7902.63	683.48	4	100	0.013	0.153	8.355
01-24	10181.04	899.46	8	100	0.010	0.123	8.661
01-25	11476.40	1117.90	1	100	0.009	0.089	7.116
01-26	9482.01	902.76	4	100	0.011	0.124	8.139
01-27	15394.54	1438.45	1	100	0.006	0.070	7.418

Table S4. Individual plasma concentrations of CoQ10 (μg/L) in the multiple dose oral administration phase—ITT population

Pt. N.	Gender	Age	Group	Multiple phase			
				Visit 2	Visit 3	Visit 4	Visit 5
01-02	M	26	A	695.91	770.46	918.24	741.59
01-05	F	43	B	770.85	1223.06	1611.59	900.83
01-06	F	41	A	1164.81	954.03	1176.15	1568.60
01-07	F	38	B	482.50	1249.19	1904.55	1093.15
01-08	F	40	A	470.60	714.82	1467.11	626.25
01-09	F	36	B	628.66	930.31	446.43	964.47
01-10	F	41	A	538.92	1348.96	1246.52	1826.85
01-11	F	49	B	673.27	906.43	650.48	796.36
01-12	F	34	A	755.60	1203.01	1036.87	1381.85
01-13	F	49	B	806.45	1033.99	917.85	2029.99
01-14	F	46	A	276.69	518.57	776.80	476.11
01-15	F	30	B	567.99	947.77	1203.67	1280.06
01-16	F	41	A	417.22	571.53	679.81	465.78
01-17	F	47	B	516.43	920.89	1116.61	1085.11
01-18	F	28	A	736.68	845.05	747.06	510.71
01-19	M	36	B	901.39	2020.62	2441.84	2309.99
01-20	F	36	A	749.91	735.65	738.58	948.23
01-21	M	42	B	1048.87	1287.08	1437.05	915.04
01-22	M	26	A	514.34	846.19	708.07	1257.38
01-23	F	50	B	672.10	885.66	961.81	1022.06
01-24	M	28	A	1292.53	1239.62	993.62	805.11
01-25	M	37	B	932.26	1753.55	861.56	1326.01
01-26	M	29	A	810.22	1005.38	766.72	729.19
01-27	M	48	B	1082.79	1554.50	1187.71	1302.28

Table S5. Individual pharmacokinetic parameters of CoQ10 in the multiple dose oral administration phase—ITT population

Subject Number	Group	AUC_t (µg/ml·h)	C_{max} (µg/l)	T_{max} (days)
01-02	A	2407.45	918.24	14
01-05	B	3670.49	1611.59	14
01-06	A	3496.89	1568.60	28
01-07	B	3941.57	1904.55	14
01-08	A	2730.36	1467.11	14
01-09	B	2173.31	964.47	28
01-10	A	3778.37	1826.85	28
01-11	B	2291.73	906.43	7
01-12	A	3308.61	1381.85	28
01-13	B	3370.06	2029.99	28
01-14	A	1671.77	776.80	14
01-15	B	3075.47	1280.06	28
01-16	A	1692.84	679.81	14
01-17	B	2838.27	1116.61	14
01-18	A	2215.81	845.05	7
01-19	B	6068.15	2441.84	14
01-20	A	2323.30	948.23	28
01-21	B	3706.09	1437.05	14
01-22	A	2440.12	1257.38	28
01-23	B	2694.55	1022.06	28
01-24	A	3282.06	1292.53	0
01-25	B	3744.25	1753.55	7
01-26	A	2541.81	1005.38	7
01-27	B	3934.75	1554.50	7
